# Long non-coding RNA PVT1/microRNA miR-3127-5p/NCK-associated protein 1-like axis participates in the pathogenesis of abdominal aortic aneurysm by regulating vascular smooth muscle cells

**DOI:** 10.1080/21655979.2021.2010384

**Published:** 2021-12-19

**Authors:** Youjin Huang, Li Ren, Jiajia Li, Haibo Zou

**Affiliations:** a Department of Vascular Surgery, Sichuan Academy of Medical Sciences and Sichuan Provincial People’s Hospital, Chengdu, Sichuan, China; b Intensive Care Unit, Sichuan Academy of Medical Sciences and Sichuan Provincial People’s Hospital, Chengdu, Sichuan, China; c Department of Hepatobiliary Surgery, Sichuan Academy of Medical Sciences and Sichuan Provincial People’s Hospital, Chengdu, Sichuan, China

**Keywords:** lncRNA PVT1, miR-3127-5p, NCKAP1L, abdominal aortic aneurysm

## Abstract

The long non-coding RNA plasmacytoma variant translocation 1 (lncRNA PVT1) has been implicated in the progression of abdominal aortic aneurysms (AAA). However, the detailed mechanism requires further analysis. Our study was aimed at interrogating the mechanism of PVT1 in an H_2_O_2_-induced AAA model *in vitro*. The expression of lncRNA PVT1, microRNA miR-3127-5p, and NCK-associated protein 1-like (NCKAP1L) was examined in AAA tissues and H_2_O_2_-treated vascular smooth muscle cells (VSMCs). Cell proliferation was assayed using Cell Counting Kit-8 (CCK8) and 5-Bromodeoxyuridine (BrdU) assays. Meanwhile, 5-Ethynyl-2′-deoxyuridine (EdU) staining was performed to assess cell apoptosis and caspase-3 activity. IL-1β and caspase-1 expression was also assessed using Western blotting to determine inflammasome activation in H_2_O_2_-treated VSMCs. Luciferase reporter assays addressed the possible interaction between miR-3127-5p and PVT1 or NCKAP1L, which was predicted by starBase analysis. PVT1 and NCKAP1L expression was elevated in AAA tissues and induced the AAA model *in vitro*, whereas miR-3127-5p showed the opposite trend. Functionally, PVT1 silencing promoted cell proliferation and reduced the apoptotic rate and inflammasome activation in H_2_O_2_-treated VSMCs. Mechanical investigation demonstrated that PVT1 acted as a sponge of miR-3127-5p to modulate NCKAP1L expression, resulting in suppression of VSMC proliferation, induction of apoptosis, and activation of inflammation. In conclusion, PVT1 participates in AAA progression through the miR-3127-5p/NCKAP1L axis and may be a promising biosignature and therapeutic target for AAA.

## Introduction

Abdominal aortic aneurysm (AAA) is a localized tumor-like dilatation of the abdominal aorta, where the abdominal aortic diameter exceeds 1.5 times the normal diameter, as a consequence of a progressively weakening of the abdominal aortic wall [1,2]. AAA has a 6% incidence in men and 1% in women, and its occurrence gradually increases with age [2,3]. Sudden rupture of the local abdominal aorta represents a major threat to AAA patients [4]. AAA reportedly has a spontaneous rupture rate of up to 50% within two years of diagnosis. When spontaneous rupture occurs, the mortality rate is higher than 90%, even with surgical therapy. More importantly, no drugs effectively inhibit the occurrence and development of AAA [5]. Recent investigations have demonstrated that the main pathological changes are abnormalities in the structure and function of the arterial wall resulting from apoptosis of VSMCs [6–8]. The VSMC phenotypic switch *in vitro* and *in vivo* has been observed in the AAA model [9]. H_2_O_2_-induced apoptosis in VSMCs has been widely used as a model for AAA research [10,11]. Therefore, a deeper understanding of the mechanism of deregulation of VSMC apoptosis and proliferation is of great importance to explore viable targets for AAA diagnosis and intervention.

Long non-coding RNAs (lncRNAs) are a class of non-coding transcripts of more than 200 nucleotides. Though of limited coding capacity, lncRNAs are regarded no longer as “noise’ but as critical regulators of gene transcription [12]. Disordered lncRNA expression is an important factor in the progression of various disorders, including AAA [13,14]. For example, recent animal experiments have demonstrated that lncRNA H19 is functionally relevant to AAA formation [15]. Li *et al*. have demonstrated that LBX2-AS1 acts as a pro-apoptotic and anti-proliferative lncRNA to induce VSMC apoptosis and hinder proliferation, thereby facilitating AAA formation [16]. The same promoting effect on AAA formation has also been found with the lncRNA GAS5 [17]. The lncRNA SENCR is poorly expressed in AAA tissues and the angiotensin II–induced AAA model, and its silencing promotes VSMC apoptosis and extracellular matrix (ECM) degradation [18]. There are many studies on the role of the lncRNA plasmacytoma variant translocation 1 (PVT1) in cancer. In most cases, lncRNA PVT1 is regarded as a tumor promoter in various malignancies which increases the malignant phenotype of tumor cells [19,20]. However, Cho *et al*. recently found that the lncRNA PVT1 promoter blunted oncogenic MYC transcription, and suppressed breast cancer tumorigenesis *in vivo* [21]. Interestingly, the inhibitory effect of lncRNA PVT1 on VSMC apoptosis and ECM disruption was also detected in a murine AAA model [22,23]. However, the underlying mechanisms remain to be fully elucidated.

The NCKAP1L gene is located on chromosome 12q13.13-q13.2, and consists of 32 exons. It encodes a member of the HEM family of tissue-specific transmembrane proteins and constitutes a part of the Scar/WAVE complex, which plays an important role in regulating cell shape. NCKAP1L is reported to interact with different immune receptors and play a role in certain immunophenotypes, and its dysregulation in immunoregulatory disorders has been examined [24,25]. Inflammatory immune responses play a critical role in AAA formation [26–28]. For example, mutations in NCKAP1L contribute to inactivation of the AKT signaling pathway, thereby resulting in T cell proliferation and immunodeficiency [24]. In two cases with NCKAP1L deficiency, Castro *et al*. found an inverted CD4/CD8 ratio, which is mainly responsible for boosting inflammation [29]. Therefore, we speculated that NCKAP1L is involved in AAA progression.

MicroRNAs, a member of non-coding RNAs, consist of approximately 20 nt, and act as important modulators in orchestrating the biofunctions of VSMCs, thus controlling AAA formation and progression [30–32]. miR-3127-5p has been reported to be deregulated in various cancers [33–35]. The inhibitory role of miR-3127-5p on tumor cell proliferation, migration, and epithelial-mesenchymal transition has been reported in different malignancies [36,37]. However, its role in AAA remains unknown.

In the present study, we first established an H_2_O_2_-induced apoptosis VSMC model to mimic AAA pathological conditions *in vitro*. Using bioinformatic analysis, we tested our hypothesis that miR-3127-5p interacts with lncRNA PVT1 and NCKAP1L, thereby playing a role in AAA. Next, we examined the function of the PVT1/miR-3127-5p/NCKAP1L axis in VSMC apoptosis and proliferation. Our findings highlight the role of lncRNA PVT1 in AAA formation, which could be helpful for exploring diagnostic and therapeutic targets against AAA.

## Methods

### Clinical tissue collection

Between January 2018 and January 2020, AAA tissues were acquired during the surgical resection of 23 AAA patients from our hospital. Normal abdominal aortic tissues were collected from subjects with physical trauma associated with AAA. Written informed consent was obtained from each participant. Ethical approval was granted by the ethical committee of the Sichuan Academy of Medical Sciences and Sichuan Provincial People’s Hospital. Surgically obtained tissues were immediately stored at −80°C for quantification of the indicated gene expression.

### Cell culture and H_2_O_2_ treatment

Human vascular smooth muscle cells (VSMCs) were purchased from the Wuhan Cell Bank of Wuhan University (Wuhan, China). All cells were cultivated in McCoy’s 5A medium containing 10% FBS and 1% penicillin-streptomycin at 37°C and 5% CO_2_. In a previous study, we developed an H_2_O_2_-induced VSMC apoptosis model to elucidate the underlying mechanism in AAA [10,11]. To establish the *in vitro* AAA model, VSMCs were incubated with 100 µM H_2_O_2_ for 48 h. Cell function assays were performed to verify the successful establishment of the H_2_O_2_-induced injury model.

### Cell transfection

Small interfering RNAs targeting lncRNA PVT1 or NCKAP1L (si-PVT1 and si-NCKAP1L) and their corresponding negative controls (si-NCs) as well as miR-3127-5p inhibitor or mimic and NCs were obtained from Hanbio, China. Transfection of H_2_O_2_-treated VSMCs was performed using Lipofectamine 2000 (Thermo Fisher Scientific, USA) following the manufacturer’s protocol. The sequences of the vectors are listed in Supplementary Table 1.

**Table 1.** The sequences of the primers in this study

Primer	Sequences
PVT1	Forward: 5′-GCCCCTTCTATGGGAATCACTA-3′
	Reverse: 5′-GGGGCAGAGATGAAATCGTAAT-3′
NCKAP1L	Forward: 5′-GTGACGGAGGCTGTTCTCTC-3′
	Reverse: 5′-TCTGAGAGTTTGCGTTGG-3′
CSF1R	Forward: 5′-GAAGGGCAGACAGAGTGTCC-3′
	Reverse: 5′-GGATTCCCTGACCATGCCAA-3′
miR-3127-5p	Forward: 5ʹ-CGGGCTTGTGGAATGGTAAGC-3’
	Reverse: 5ʹ-CTGTCAGCTTCCCATTCC-3’
GAPDH	Forward: 5ʹ-CGCTCTCTGCTCCTCCTGTTC-3’
	Reverse: 5ʹ-ATCCGTTGCTCCGACCTTCAC-3’
U6	Forward: 5ʹ-GCTTCGGCAGCACATATACTAAAAT-3’
	Reverse: 5ʹ-CGCTTCACGAATTTGCGTGTCAT-3’

### Real time-quantitative PCR (RT-qPCR)

TRIzol reagent was used to isolate RNA from the clinical samples and from cells. cDNA was synthesized from RNA using the Prime Script RT reagent kit (Takara, Japan). Quantification of cDNA was conducted by quantitative PCR (qPCR) analysis using an Applied Biosystems TaqMan kit (Thermo Fisher Scientific, USA) on an AB7500 instrument (Applied Biosystems, UK). The data were analyzed by the 2^−∆∆Ct^ method [38] with normalization to U6 or GAPDH expression. The primers used are listed in Table 1.

### Assessment of cell proliferation

For Cell Counting Kit-8 (CCK8) assays, a CCK8 kit (Beyotime, China) was used according to a previous study [16]. Transfected H_2_O_2_-treated VSMCs (1 × 10^3^ cells/well) were plated in 96-well plates. After maintenance for 1, 3, and 5 days, the cells were exposed to 10 µL CCK-8 reagent for 30 min. The plates were assayed using a microplate reader at a wavelength of 450 nm.

For 5-Bromodeoxyuridine (BrdU) assays, a BrdU Cell Proliferation Assay Kit (Frdbio, China) was used according to the manufacturer’s instructions. In brief, transfected H_2_O_2_-treated VSMCs (1 × 10^3^ cells/well) were seeded into 96-well plates. When the cell confluence reached 60%, 20 μM of BrdU solution was added for 12 h. After the cells were exposed to cell fixation buffer for 30 min, they were continuously treated with denaturing solution for an additional 30 min. Subsequently, BrdU antibodies were added to the cells. One hour later, a FACSLyrics flow cytometer was used to quantify labeled cells.

### Evaluation of apoptosis

First, a Caspase-3 activity assay kit (Leagene, China) was used according to the manufacturer’s instructions. Briefly, the cells were plated in 96-well plates. After 48 h of cultivation, cells were lysed with caspase lysis buffer and then reacted with 10 µL 2 mM Ac-DEVD-pNA at 37°C for 2 h. The resulting p-nitroanilide (pNA) was measured using an ELISA plate reader at 405 nm.

Apoptosis was also evaluated using terminal deoxynucleotidyl transferase dUTP nick end labeling (TUNEL) and Hoechst staining according to a previous study [16]. Transfected H_2_O_2_-treated VSMCs were plated in 6-well plates containing coverslips. When the cells reached 75% confluence, they were deparaffinized and rehydrated following treatment with 20 µg/mL proteinase K (Qiagen, Netherlands). After repeated rinsing, the cells were labeled with TUNEL agents and post-counterstained with Hoechst (2 µg/mL). Cells were observed and photographed using a fluorescent microscope.

### Western blotting

Cell pellets were lysed in cold radioimmunoprecipitation assay buffer. After microcentrifugation, the supernatant was collected for protein quantification using a Pierce BCA Protein Assay Kit (Thermo Scientific, USA). A total of 15 μg of protein was loaded onto 10% acrylamide gels for sodium dodecyl sulfate–polyacrylamide gel electrophoresis and run at 150 V until the dye front reached the bottom of the gels. A semi-dry transfer system was used to transfer the gel to the polyvinylidene difluoride membrane. Next, the membrane was blocked with 5% bovine serum albumin (Sigma, USA) at room temperature following labeling with anti-NCKAP1L antibody (Cat# HPA039490, 1:1000; Sigma, USA), anti-SAB2108668 antibodies (Cat#SAB2108668, 1:1000; Sigma, USA) overnight at 4°C. After treatment with horseradish peroxidase-conjugated mouse and rabbit secondary antibodies for 1 h at room temperature, ECL Western blotting Detection System (Fisher Scientific, USA) was used to detect the bands [16].

### Dual-Luciferase reporter assay

Luciferase reporter vectors were constructed as described previously [39]. Briefly, the constructed wild-type (WT) and mutant (MUT) luciferase reporter vectors were simultaneously delivered into VSMCs. Forty-eight hours post-transfection, luciferase activity was detected using the Dual-Glo luciferase assay kit (Promega, USA).

### Statistical analysis

GraphPad Prism 6 was used to process statistics. Unpaired Student’s *t*-tests and ANOVA were used to compare the data from two groups or multiple groups, respectively. Pearson correlations were performed. *P* < 0.05 was considered significant.

## Results

Our study aimed to explore the function of the lncRNA PVT1/miR-3127-5p/NCKAP1L axis in AAA. After performing bioinformatics analysis and cell function experiments, we confirmed that lncRNA PVT1 silencing promoted cell proliferation and reduced the apoptotic rate and inflammasome activation in H_2_O_2_-treated VSMCs by sponging the miR-3127-5p/NCKAP1L axis.

### Identification of the lncRNA PVT1/miR-3127-5p/NCKAP1L axis in AAA

**Figure 1.**
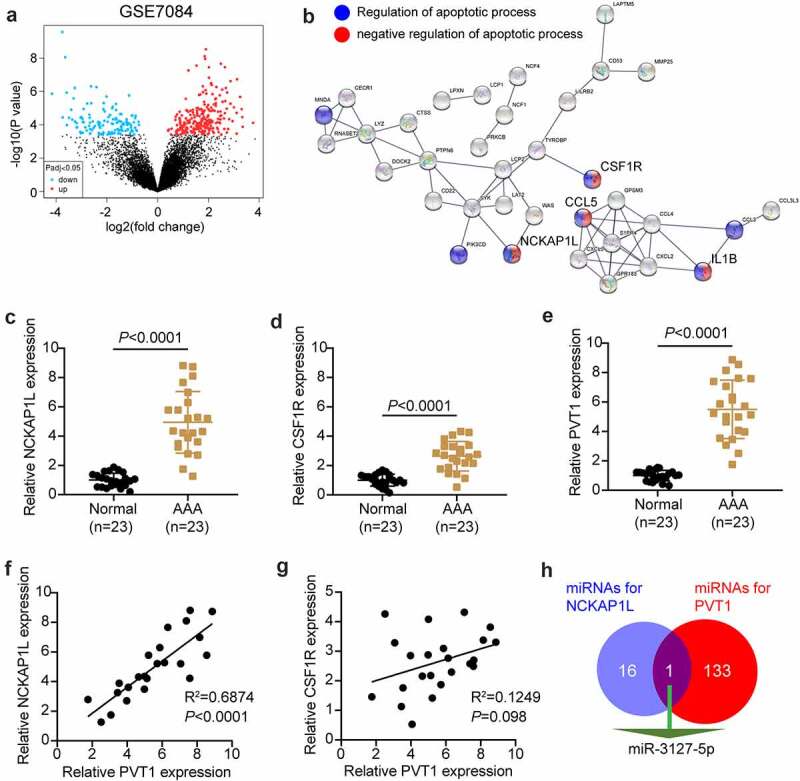
**lncRNA PVT1/miR-3127-5p/NCKAP1L axis might be associated with AAA**. (a) 335 differentially expressed genes (DEGs) from GSE7084 were screened out with adj.P value<0.05. (b) STRING was used to enrich the key biological processes for upregulated DEGs. (c-d) The expression of NCKAP1L and CSF1R in our collected AAA samples. (e) The expression of lncRNA PVT1 in our collected AAA samples. (f) Pearson correlation coefficient between lncRNA PVT1expression and NCKAP1L expression. (g). Pearson correlation coefficient between lncRNA PVT1expression and CSF1R expression. (h) miR-3127-5p predicted by starBase could bind to NCKAP1L and PVT1

The GSE7084 dataset from Gene Expression Omnibus DataSets includes six AAA and seven non-AAA samples. The 335 differentially expressed genes (DEGs) were screened out with an adjusted P value < 0.05 (Figure 1a). With log fold change (logFC) > 2, 71 upregulated DEGs in AAA samples were uploaded to STRING for biological process enrichment. The results showed that NCKAP1L, CSF1R, CCL5, and IL1B were associated with both the positive and negative regulation of apoptosis (Figure 1b). Due to the clear effect of CCL5 and IL1β on AAA [40,41], NCKAP1L and CSF1R attracted our attention. In our RT-qPCR analysis, NCKAP1L was upregulated more significantly than CSF1R in our AAA samples (Figure 1c, d). lncRNA PVT1 was reported to promote VSMC apoptosis and extracellular matrix disruption in the AAA model [22], but its regulatory mechanism is unclear. RT-qPCR analysis showed that lncRNA PVT1 was highly expressed in AAA samples (Figure 1e). Following Pearson’s test analysis, NCKAP1L showed a tighter negative correlation with PVT1 than CSF1R (figure 1f, g). Therefore, NCKAP1L was identified as the interested gene. Next, we attempted to identify a miRNA that could connect PVT1 and NCKAP1L. StarBase was used to predict this, showing that miR-3127-5p was the only miRNA that connected PVT1 and NCKAP1L (Figure 1h).

### LncRNA PVT1 is upregulated in AAA tissues and targets miR-3127-5p

**Figure 2.**
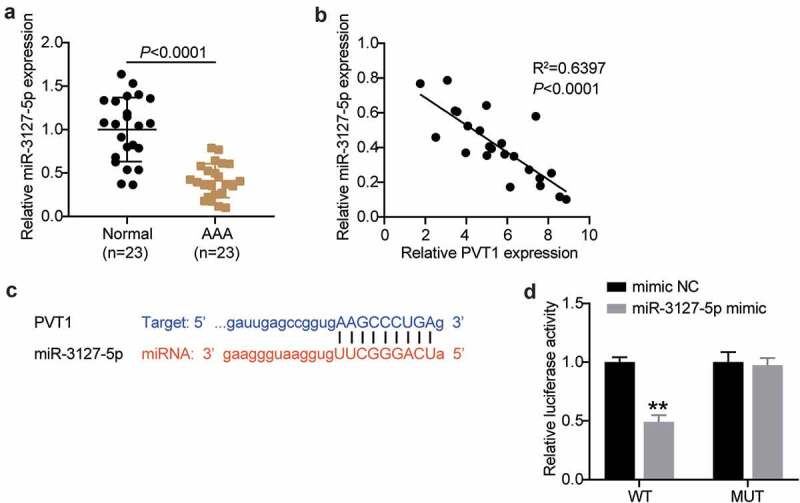
lncRNA PVT1 is upregulated in AAA tissues and targets to miR-3127-5p. (a) RT-qPCR analysis examined the miR-3127-5p expression in in AAA tissues. (b) Pearson correlation analysis of lncRNA PVT1 expression with miR-3127-5p expression. (c) A binding site of lncRNA PVT1 to miR-3127-5p was presented by using starBase. (d) Binding of lncRNA PVT1 to miR-3127-5p confirmed by dual-luciferase reporter assay, vs. control, *P < 0.05,**P < 0.001

Considering the involvement of lncRNA PVT1 and miR-3127-5p in AAA progression by bioinformatics analysis, we further examined their expression in AAA tissues. As shown in Figure 2a, AAA tissues showed poor expression of miR-3127-5p. Interestingly, after Pearson’s analysis, we found that lncRNA PVT1 and miR-3127-5p were negatively correlated (Figure 2b). Therefore, we employed starBase analysis and luciferase reporter assays to further identify their relationship. As shown in Figure 2c, lncRNA PVT1 carried nine bases complementary to miR-3127-5p. Furthermore, the miR-3127-5p mimic decreased lncRNA PVT1-WT-driven luciferase activity while showing no impact on lncRNA PVT1-MUT-driven activity (Figure 2d). Therefore, we propose that lncRNA PVT1 targets miR-3127-5p and interferes with AAA progression.

### Establishment of the VSMC injury model for AAA using H_2_O_2_

**Figure 3.**
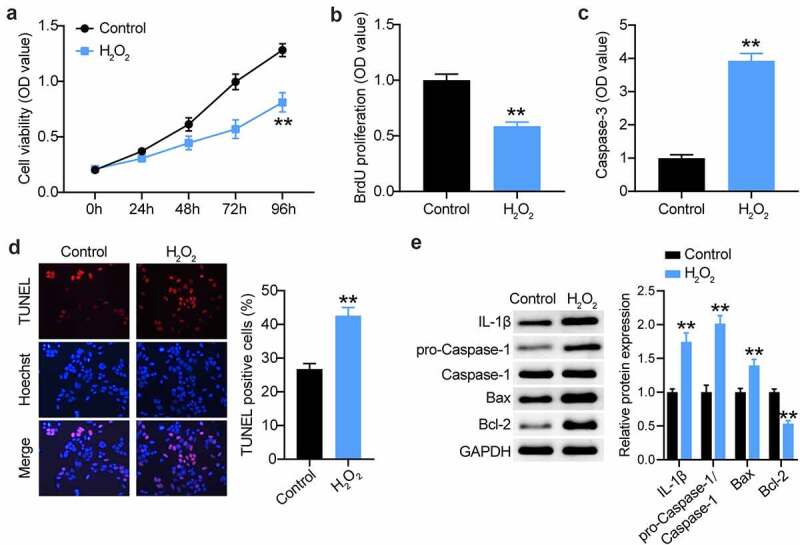
Establishment of VSMCs injury model for AAA using H2O2. (a and b) CCK-8 and EdU assays were used to detect the cell proliferation in VSMCs treated with H2O2 or not. (c and d) Caspase-3 activity, TUNEL and flow cytometry assays were performed to measure cell apoptosis in VSMCs treated with H2O2 or not, (e) Western blot analysis of IL-1, caspase-1, pro-caspase-1, Bax and Bcl-2 in VSMCs treated with H2O2 or not, vs. control, *P < 0.05,**P < 0.001

The H_2_O_2_-treated VSMC model is an *in vitro* model for AAA research [10,42]. Therefore, we first treated VSMCs with 100 µM H_2_O_2_ for 48 h. As shown in Figure 3a, a proliferative defect was observed in the VSMCs after H_2_O_2_ treatment, as evidenced by CCK8 assays. Likewise, BrdU proliferation assays also confirmed the reduction in the proliferation of VSMCs after H_2_O_2_ treatment (Figure 3b). Not surprisingly, apoptosis assessment showed that H_2_O_2_ exposure triggered VSMC apoptosis, which was evidenced by the increased caspase-3 activity and TUNEL-positive cell rate (Figure 3c, d). In general, H_2_O_2_ is implicated in many inflammatory cascades. Therefore, we assessed caspase-1 and IL-1β during VSMC induction. As shown in Figure 3e, caspase-1 and IL-1β expression was augmented in H_2_O_2_-triggered VSMCs, coupled with increased Bax expression and decreased Bcl-2 expression. Therefore, the *in vitro* AAA model was used for the subsequent assays.

### MiR-3127-5p inhibitor abrogates the effect of lncRNA PVT1 silencing on proliferation and apoptosis of H_2_O_2_-treated VSMCs

**Figure 4.**
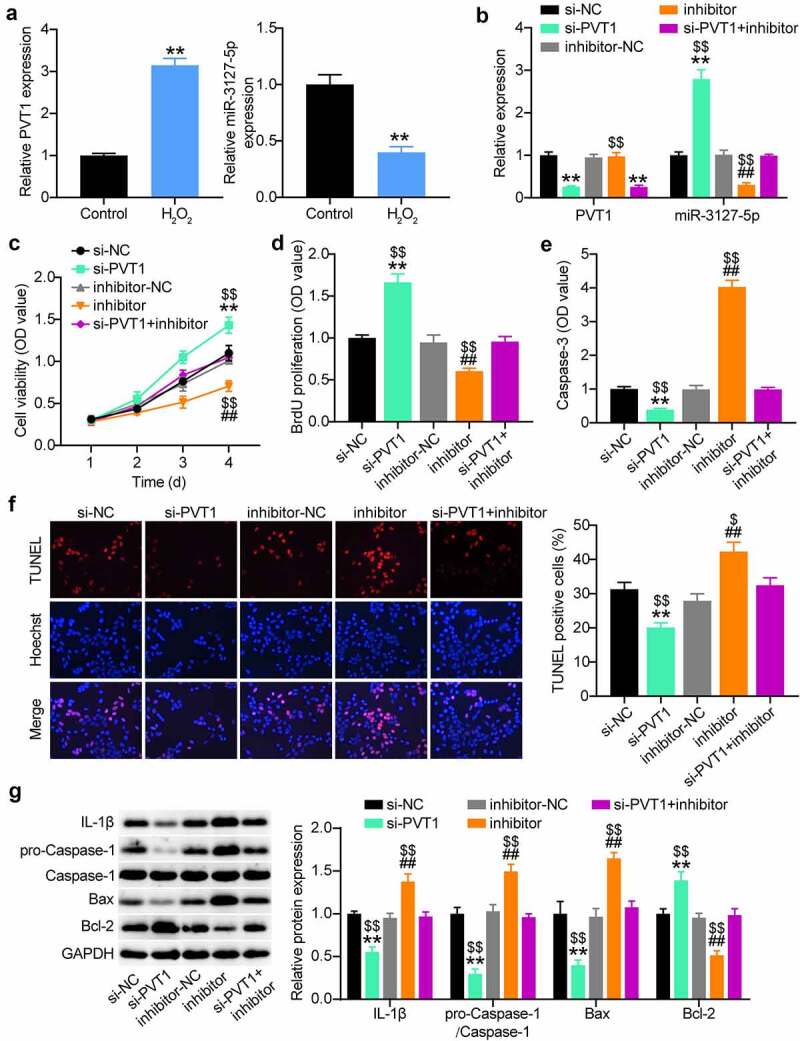
miR-3127-5p inhibitor abrogates the effect of lncRNA PVT1 silence on proliferation and apoptosis of H2O2-treating VSMCs

To identify the sponge activity of lncRNA PVT1 to miR-3127-5p during AAA progression, we first examined their expression in H_2_O_2_-treated VSMCs. The Quantitative reverse transcription PCR (RT-qPCR) analysis demonstrated the upregulation of lncRNA PVT1 and downregulation of miR-3127-5p (Figure 4a, b). When H_2_O_2_-treated VSMCs were transfected with si-PVT1, a significant enhancement of the miR-3127-5p expression level was detected; however, this phenomenon was reversed by co-transfection of miR-3127-5p inhibitor (Figure 4c). CCK8 and BrdU proliferation assays demonstrated that anti-miR-3127-5p treatment increased the proliferation of H_2_O_2_-treated VSMCs, and an abrogation of the increased proliferation by PVT1 silencing (Figure 4d, e). Interestingly, anti-miR-3127-5p treatment increased caspase-3 activity, which was weakened by si-PVT1 in H_2_O_2_-treated VSMCs. In addition, the TUNEL assay further demonstrated the nullification of increased apoptosis in H_2_O_2_-treated VSMCs transfected with si-PVT1 and miR-3127-5p inhibitor (figure 4f). The miR-3127-5p inhibitor aggravated the inflammatory cascades caused by H_2_O_2_, supporting the notion that the higher expression of caspase-1 and IL-1β was abrogated by additional transfection with si-PVT1 (Figure 4g). Therefore, lncRNA PVT1 silencing can upregulate miR-3127-5p and attenuate H_2_O_2_ damage in VSMC proliferation.

(a and b) The lncRNA PVT1 and miR-3127-5p expression in H2O2-treating VSMCs by RT-qPCR. (c) H2O2-treating VSMCs was transfected with si-PVT1, si-NC, miR-3127-5p inhibitor, miR-3127-5p inhibitor-NC, and si-PVT1+ miR-3127-5p inhibitor. After 48 h, RT-qPCR were perfomred to detect the transfection efficiency. (c) The transfected H2O2-treating VSMCs underwent CCK8 assays to test the cell proliferation. (d) Brudu assays were conducted to examine the proliferation of transfected H2O2-treating VSMCs. (e) Evaluation of caspase-3 activity in transfected H2O2-treating VSMCs. (f) TUNEL and flow cytometry assays were performed to measure cell apoptosis of VSMCs. (g) Western blot analysis of IL-1, caspase-1, pro-caspase-1, Bax and Bcl-2 in VSMCs. P < 0.05,** P < 0.001, vs.Si-NC; # P < 0.05, ## P < 0.001, vs.inhibitor-NC; $ P < 0.05, $$ P < 0.001, vs.si-PVT1+ inhibitor.

### Identification of NCKAP1L as an effector of lncRNA PVT1/miR-3127-5p competing endogenous RNA activity

**Figure 5.**
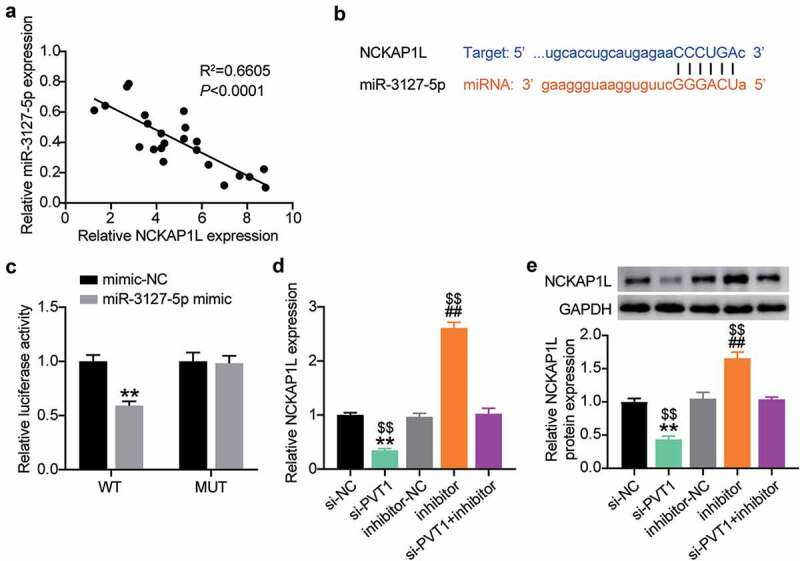
Identification of NCKAP1L as an effector of lncRNA PVT1/miR-3127-5p ceRNA activity

Next, we analyzed the interaction between miR-3127-5p and NCKAP1L. As shown in Figure 5a, a negative correlation between their expression levels was detected in the AAA tissues (Figure 5a). The starBase prediction analysis revealed that the 3′-UTR of NCKAP1L contained a binding seed region for miR-3127-5p (Figure 5b). The physical binding between them was demonstrated by luciferase reporter assays showing the reduction of 3′-UTR NCKAP1L-mediated luciferase activity in VSMCs transfected with miR-3127-5p (Figure 5c). Since PVT1 has competing endogenous RNA (ceRNA) activity for the miR-3127-5p/NCKAP1L axis, we further tested the impact of co-transfection of the miR-3127-5p inhibitor and si-PVT1 to H_2_O_2_-treated VSMCs on NCKAP1L expression. As expected, the loss of miR-3127-5p increased NCKAP1L expression, while the loss of PVT1 produced an opposite effect on NCKAP1L expression (Figure 5c). More importantly, the reduction in NCKAP1L expression caused by PVT1 silencing in H_2_O_2_ treated VSMCs was reversed by the addition of miR-3127-5p inhibitor (Figure 5d, e). Therefore, PVT1 can promote NCKAP1L expression via miR-3127-5p target inhibition.

(a) Pearson correlation coefficient between miR-3127-5p expression and NCKAP1L expression. (b) Binding sequence of miR-3127-5p in NCKAP1L 3′ UTR predicted by starBase. (c) Normalized luciferase activity of H2O2-treating VSMCs nucleofected with the indicated miRNA mimics and 3′UT NCKAP1Lluciferase reporter vectors. (d) H2O2-treating VSMCs were transfected si-PVT1, si-NC, miR-3127-5p inhibitor, miR-3127-5p inhibitor NC, si-PVT1+ miR-3127-5p inhibitor. After 48 h, RT-qPCR were performed to examine NCKAP1LmRNA level. (e) Western blots were conducted to detect the NCKAP1Lprotein expression in cells (d) P < 0.05,** P < 0.001, vs.Si-NC; # P < 0.05, ## P < 0.001, vs.inhibitor-NC; $ P < 0.05, $$ P < 0.001, vs.si-PVT1+ inhibitor.

### NCKAP1L silencing reverses the effect of miR-3127-5p inhibitor on proliferation and apoptosis of H_2_O_2_-treating VSMCs

**Figure 6.**
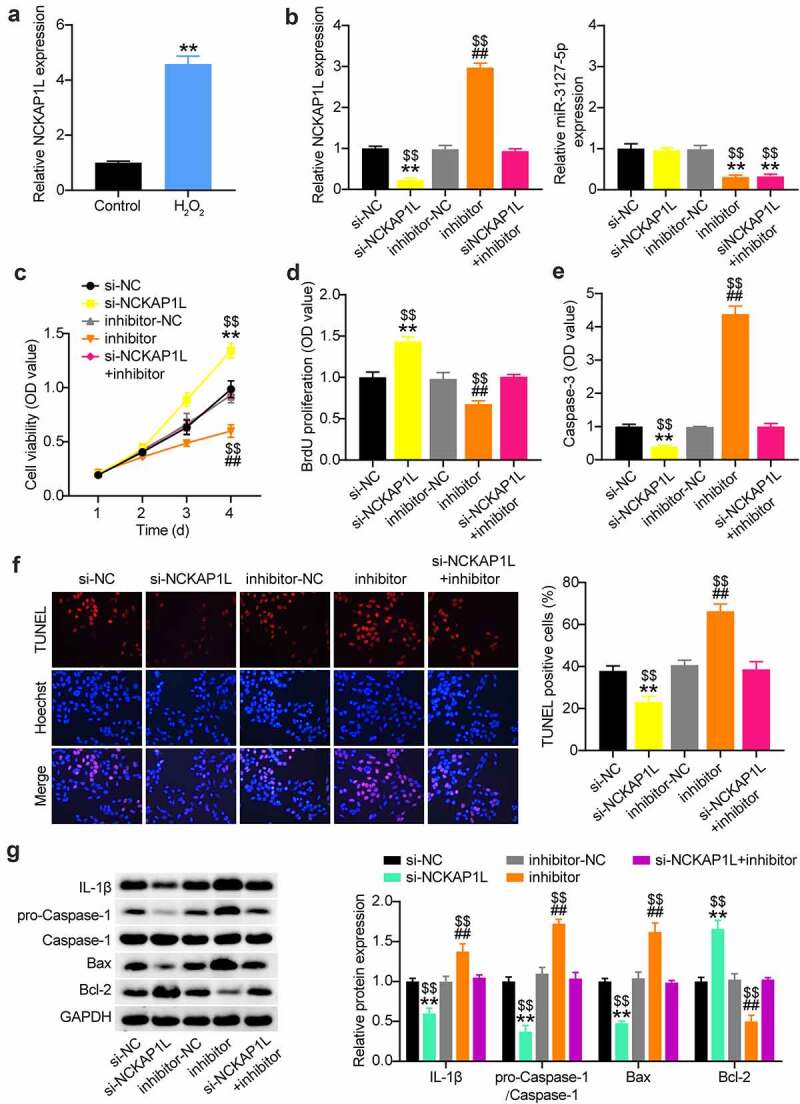
NCKAP1L silence recuses the effect of miR-3127-5p inhibitor on proliferation and apoptosis of H2O2-treating VSMCs

The elevation of NCKAP1L expression in H_2_O_2_-treated VSMCs (Figure 6a) triggered our interest in exploring whether NCKAP1L is critical for the lncRNA PVT1/miR-3127-5p axis in AAA. We simultaneously introduced si-NCKAP1L and miR-3127-5p inhibitor into H_2_O_2_-treated VSMCs. RT-qPCR analysis demonstrated that the miR-3127-5p inhibitor rescued the downregulation of NCKAP1L resulting from si-NCKAP1L (Figure 6b). Assessment of cell proliferation by CCK8 and BrdU assays revealed that NCKAP1L knockdown accelerated the proliferation of H_2_O_2_-treated VSMCs; however, this enhanced proliferation was offset by the miR-3127-5p inhibitor (Figure 6c, d). Meanwhile, NCKAP1L knockdown blunted apoptosis of H_2_O_2_-treated VSMCs, which was rescued by the miR-3127-5p inhibitor, as evidenced by caspase-3 activity and TUNEL assays (Figure 6e, f). Silencing of NCKAP1L reduced the expression of caspase-1, IL-1β, and Bax while increasing Bcl-2 expression. This suggests that its silencing mitigates the inflammatory status of VSMCs caused by H_2_O_2_, which was offset by the miR-3127-5p inhibitor (Figure 6g). Therefore, miR-3127-5p downregulation increased NCKAP1L expression and controlled the proliferation and apoptosis of H_2_O_2_-treated VSMCs.

(a) RT-qPCR analysis of NCKAP1L expression in H2O2-treating VSMCs. (b) si-NC, si-NCKAP1L, miR-3127-5p inhibitor, miR-3127-5p inhibitor NC, miR-3127-5p inhibitor+si-NCKAP1L were introduced into H2O2-treating VSMCs for 48 h. The RT-qPCR was conducted to analyze the miR-3127-5p and NCKAP1L expression. (c and d). CCK-8 and EdU assays were used to detect the cell proliferation ability of VSMCs in different groups. (e and f). Caspase-3 activity, TUNEL and flow cytometry assays were performed to measure cell apoptosis of VSMCs in in different groups. (g) Western blot analysis of IL-1, caspase-1, pro-caspase-1, Bax and Bcl-2 in VSMCs. * P < 0.05, ** P < 0.001, vs.Si-NC; # P < 0.05, ## P < 0.001, vs.inhibitor-NC; $P < 0.05, $$P < 0.001, vs.si-NCKAP1L+inhibitor.

## Discussion

Recently, lncRNA PVT1 was found to be highly expressed in AAA patients, and PVT1 silencing attenuated Ang II–induced AAA-associated alterations in an Ang II–induced murine model. Therefore, we explored the molecular mechanism underlying PVT1-mediated AAA progression in the H_2_O_2_ induced VSMCs injury model. In an *in vitro* model of AAA, PVT1 knockout not only inhibited VSMC apoptosis and ECM disruption, but also suppressed VSMC pyroptosis and inflammation [22,23]. We also found that the high expression of PVT1 in AAA tissues and PVT1 silencing increased VSMC proliferation, blunted the apoptosis rate, and alleviated inflammatory events in the H_2_O_2_-induced apoptotic model of VSMCs. More importantly, we found that PVT1 sequesters miR-3127-5p and consequently upregulates NCKAP1L expression, inhibits cell proliferation, and triggers inflammatory events, resulting in AAA development. Our findings suggest that targeting PVT1 may be a druggable approach for AAA management.

MicroRNAs are also a member of non-coding RNAs. Unlike other non-coding RNAs, miRNAs are approximately 20 nt in length. Mounting evidence has demonstrated that miRNAs act as important modulators to orchestrate the biofunctions of VSMCs, thus controlling AAA formation and progression. For example, miR-144-5p inhibits Ang II–induced aortic dilatation and attenuates the high expression of M1 macrophage markers, in which aberrant hyperactivation contributes to AAA formation [43]. MiR-33-5p is abundantly expressed in AAA tissues, and its silencing can activate PI3K/Akt signaling cascades and consequently trigger the inflammatory response, and thus be involved in AAA pathology [44]. In the present study, we identified a novel miRNA downregulated in AAA tissues and H_2_O_2_-induced VSMCs, miR-3127-5p. Furthermore, anti-miR-3127-5p treatment reduced the proliferative rate, elevated the apoptotic rate in H_2_O_2_-induced VSMCs, and triggered inflammatory events. Therefore, miR-3127-5p may attenuate the VSMCs damage induced by H_2_O_2_. Crosstalk of non-coding RNAs plays an important role in the regulation of AAA progression. Consistent with our bioinformatic analysis results, starBase analysis and luciferase reporter assays validated the extensive pairing complementarity between miR-3127-5p and lncRNA PVT1. Furthermore, si-PVT1 transfection completely recovered miR-3127-5p expression in miR-3127-5p-transfected H_2_O_2_-induced VSMCs. The interplay was further validated by the negative correlation between PVT1 and miR-3127-5p in AAA tissues. Cell function assays also showed that the miR-3127-5p inhibitor abrogated the inhibition of si-PVT1 on apoptosis of H_2_O_2_-induced VSMCs and restored the reduced inflammatory response in H_2_O_2_-induced VSMCs by PVT1 silencing. Hence, PVT1 functions as a ceRNA for miR-3127-5p to inhibit the proliferative phenotypes of H_2_O_2_-induced VSMCs, aggravating AAA progression.

For decades, lncRNAs have been shown to function as sponges of miRNAs and interfere with the suppression of miRNAs of their target mRNAs [13]. As indicated by our bioinformatic analysis, we found that NCKAP1L mRNA interacted with the lncRNA PVT1/miR-3127-5p axis. Clinically, NCKAP1L was found to be robustly expressed in AAA tissues, and in the H_2_O_2_-induced VSMC model, increased expression of NCKAP1L was also observed. Although the role of NCKAP1L in immunology and the inflammatory response has been acknowledged [24,29], its role in AAA remains unclear. We found augmented proliferation and reduced apoptosis in H_2_O_2_-induced VSMCs following NCKAP1L silencing. These data suggest that NCKAP1L might aggravate AAA progression by increasing VSMC apoptosis. As for the predicted ceRNA activity, luciferase reporter assays supported the physical binding of miR-3127-5p to NCKAP1L mRNA. Interestingly, PVT1 silencing offset the increased endogenous NCKAP1L expression caused by ant-miR-3127-5p treatment in H_2_O_2_-induced VSMCs. The negative correlation between miR-3127-5p and NCKAP1L further reinforced the ceRNA network constituted by NCKAP1L, miR-3127-5p, and PVT1. Proliferation and apoptosis assays demonstrated that anti-miR-3127-5p treatment abrogated the effect of NCKAP1L silencing on proliferation and apoptosis. Additionally, NCKAP1L silencing abrogated the increased inflammatory event caused by the miR-3127-5p inhibitor. These data suggest that the PVT1/miR-3127-5p/NCKAP1L axis suppresses proliferation and induces apoptosis and inflammation in H_2_O_2_-induced VSMCs, consequently boosting AAA progression. Notably, Xiong *et al*. also reported that the promotion of AAA by PVT1 is mediated by the miR-26a/KLF4 axis through the PI3K/AKT signaling pathway [23]. In contrast, NCKAP1L loss blunts AKT phosphorylation and modulates immunodeficiency [24]. Hence, it has been speculated that PVT1 is an active player in AAA progression by its ceRNA activity in modulating the AKT signaling pathway. Consequently, further research will focus on the PVT1-mediated complex regulatory network in AAA progression.

Our study had several limitations. First, the low number of tissue samples might have led to a selective bias. *In vivo* experiments were not performed to support the *in vitro* findings. In addition, our study focused only on the PVT1/miR-3127-5p/NCKAP1L axis in AAA progression. In the future, we will continue to explore the underlying mechanisms downstream of NCKAP1L.

## Conclusion

In conclusion, our data indicate that lncRNA PVT1 is involved in AAA progression by sequestering miR-3127-5p and enhancing the expression of its target, NCKAP1L, indicating that the PVT1/miR-3127-5p/NCKAP1L axis could be an ideal drug target for AAA prevention.

## Supplementary Material

Supplemental Material

## Data Availability

The datasets used and/or analyzed during the current study are available from the corresponding author on reasonable request (haibozou9@163.com).

## Highlights

- LncRNA PVT1 is upregulated in AAA tissues.
- PVT1 silencing promotes cell proliferation and reduces the apoptotic rate and inflammasome activation of H_2_O_2_-treated VSMCs.
- PVT1 can sponge miR-3127-5p to upregulate NCKAP1L expression in VSMCs.
