# Supplementary material for: Long non-coding RNA PVT1/microRNA miR-3127-5p/NCK-associated protein 1-like axis participates in the pathogenesis of abdominal aortic aneurysm by regulating vascular smooth muscle cells
Source: Bioengineered. 2021 Dec 19;12(2):12583–96. doi: 10.1080/21655979.2021.2010384 (PMC8810122; doi:10.1080/21655979.2021.2010384)
Supplement: Supplemental Material [file KBIE_A_2010384_SM9455.zip › supplementary/Supplementary table 1.docx]

Supplementary table 1. The sequences of vectors used in this study.

| **Vector** | **Sequences** |
| --- | --- |
| **Si-PVT1** | 5′-GAGCUGCGAGCAAAGAUGU-3′ |
| **Si-NCKAP1L** | 5′-GGAGUAACGUACAAGUAAA-3′ |
| **miR-3127-5p mimic** | 5′-AUCAGGGCUUGUGGAAUGGGAAG-3′ |
| **miR-3127-5p inhibitor** | 5'- AUCAGGGCUUGUGGAAUGGGAAG-3' |
